# Ambient PM2.5 and Annual Lung Cancer Incidence: A Nationwide Study in 295 Chinese Counties

**DOI:** 10.3390/ijerph17051481

**Published:** 2020-02-25

**Authors:** Huagui Guo, Weifeng Li, Jiansheng Wu

**Affiliations:** 1 Department of Urban Planning and Design, The University of Hong Kong, Hong Kong 999077, China; huaguiguo@hku.hk; 2 Shenzhen Institute of Research and Innovation, The University of Hong Kong, Shenzhen 518057, China; 3 Key Laboratory for Urban Habitat Environmental Science and Technology, Shenzhen Graduate School, Peking University, Shenzhen 518055, China; wujs@pkusz.edu.cn; 4 Key Laboratory for Earth Surface Processes, Ministry of Education, College of Urban and Environmental Sciences, Peking University, Beijing 100871, China

**Keywords:** PM2.5, lung cancer incidence, long-term lag effect, China

## Abstract

Most studies have examined PM2.5 effects on lung cancer mortalities, while few nationwide studies have been conducted in developing countries to estimate the effects of PM2.5 on lung cancer incidences. To fill this gap, this work aims to examine the effects of PM2.5 exposure on annual incidence rates of lung cancer for males and females in China. We performed a nationwide analysis in 295 counties (districts) from 2006 to 2014. Two regression models were employed to analyse data controlling for time, location and socioeconomic characteristics. We also examined whether the estimates of PM2.5 effects are sensitive to the adjustment of health and behaviour covariates, and the issue of the changing cancer registries each year. We further investigated the modification effects of region, temperature and precipitation. Generally, we found significantly positive associations between PM2.5 and incidence rates of lung cancer for males and females. If concurrent PM2.5 changes by 10 μg/m^3^, then the incidence rate relative to its baseline significantly changes by 4.20% (95% CI: 2.73%, 5.88%) and 2.48% (95% CI: 1.24%, 4.14%) for males and females, respectively. The effects of exposure to PM2.5 were still significant when further controlling for health and behaviour factors or using 5 year consecutive data from 91 counties. We found the evidence of long-term lag effects of PM2.5. We also found that temperature appeared to positively modify the effects of PM2.5 on the incidence rates of lung cancer for males. In conclusion, there were significantly adverse effects of PM2.5 on the incidence rates of lung cancer for both males and females in China. The estimated effect sizes might be considerably lower than those reported in developed countries. There were long-term lag effects of PM2.5 on lung cancer incidence in China.

## 1. Introduction

Air pollution has been a worldwide environmental problem and major risk to human health. Fine particular matter pollution (PM2.5) and its detrimental health effects have raised great public health concerns around the world, not only in China. According to the International Agency for Research on Cancer Monographs on the Evaluation of Carcinogenic Risks to Humans, PM2.5 has become a carcinogenic factor for lung cancer [1,2]. It has been estimated that outdoor air pollution including PM2.5 has caused 4.2 million premature deaths per year in the world, with the greatest number in South-East Asia and the Western Pacific regions and with 6% of deaths caused by lung cancer [3]. Currently, trachea, bronchus and lung cancers represent the majority of cancer incidences in China, with the age-adjusted incidence rate of 36.54 per 100,000 in 2014 [4]. However, despite some efforts examining PM2.5 or PM10 effects on lung cancer mortality [5,6,7,8], the associations between exposure to PM2.5 and lung cancer incidence were not well and comprehensively understood in China.

Most research on PM2.5-associated lung cancer incidence were conducted in developed countries, where health outcome data are more readily available. As published in the International Agency for Research on Cancer Monographs (volume 109) on the Evaluation of Carcinogenic Risks to Humans, biologically, the exposure to outdoor air pollution, which includes the ambient particulate matter, would lead to increased cancer risks in humans through the elevations in genetic damage, such as cytogenetic abnormalities, altered gene expression and mutations in both somatic and germ cells [1,2]. Empirically, the substantial body of studies in developed countries suggested a significant and positive association between PM2.5 exposure and lung cancer incidence. In particular, the 17 prospective European cohort studies that were performed in 12 European areas (one of the largest and most informative studies) suggested that a 5 μg/m^3^ increment in annual exposure to PM2.5 was correlated with the elevation of hazard ratio of lung cancer incidence by 1.18 [9]. Even restricting the study population to those who had never smoked, long-term exposure to PM2.5 was still statistically and positively associated with the risk of lung cancer mortality [10]. Other nationwide studies in western countries further enhance the evidence of significant effects of PM2.5 exposure on lung cancer incidence [11,12,13]. However, these nationwide or multisite long-term estimations from developed countries might not be applicable to China. There are significant differences in the air pollution level between China and Western countries. More specifically, the annual medium concentration of PM2.5 in China was 54 μg/m^3^, which is more than four times higher than that in Western countries (e.g., 12 μg/m^3^ for the United Kingdom, 8 μg/m^3^ for the United States) [3], which highlights the significance of examining the association between PM2.5 and lung cancer incidence in other places, such as China.

In comparison, studies that estimate PM2.5 effects on lung cancer incidence in developing countries are quite limited. Overall, these limited studies indicated a significant and adverse effect of PM2.5 exposure on lung cancer incidence. One of the representative studies is a nationwide time-series study with lung cancer incidence data from 75 Chinese cancer registries from 1990 to 2009. Using the spatial age-period-cohort model, the authors found that there were positive associations between 2 year average PM2.5 exposure and lung cancer incidence for both males and females [14]. Similarly, as suggested in a multisite study of 72 Chinese cities, both the concurrent and previous 8 year annual PM2.5 concentrations were significantly associated with male lung cancer incidence [15]. However, these multisite or nationwide studies that estimate PM2.5 effects on lung cancer incidence are still quite limited in developing countries. In addition, to have an in-depth understanding of particulate matter effects on lung cancer outcome, especially for lung cancer incidence, further research is needed according to previous study findings. First, these studies either focused on single site [16] or only urban areas in China [7,15,17], which highlights the necessity for nationwide examinations that include both the urban and rural areas in China. Second, there are relatively few studies examining the long-term effects of particulate matter [6,7,18], especially for the long-term lag response of lung cancer incidence to PM2.5. Third, most of these studies paid attention to the examination of PM2.5-associated lung cancer mortalities [5,7,8,19], while PM2.5-associated lung cancer incidences were not well and comprehensively examined.

To fill the gaps above, we conducted a nationwide study to estimate the effects of PM2.5 exposure on annual incidence rates of trachea, bronchus and lung cancers in China, using the population-based health outcome data collected from 295 Chinese cancer registries from 2006 to 2014. We determined the estimates using two regression models with controls for location, time and socioeconomic characteristics. We further investigated whether the effects were modified according to the region, temperature and precipitation.

## 2. Materials and Methods

### 2.1. Study Area

**Figure 1:**
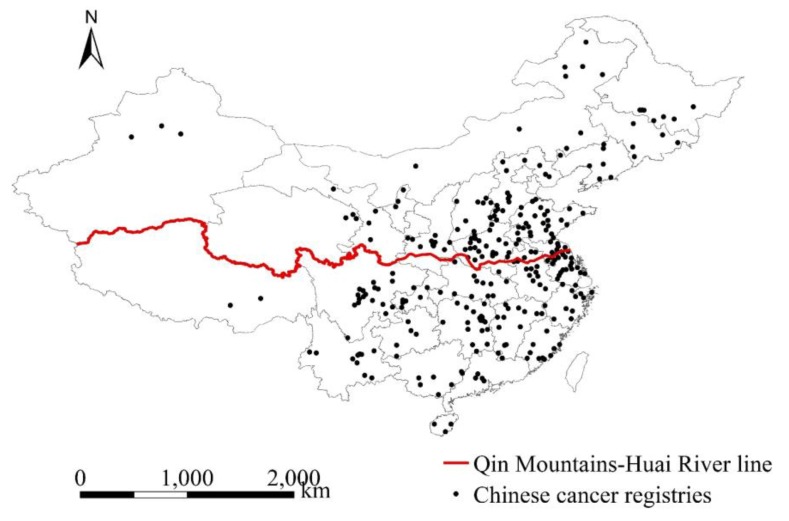
Locations of Chinese cancer registries.

The study area covered 295 county-level cancer registries in China, which included 222 counties (rural registries) and 73 districts (urban registries). The number of registries covered by the Chinese Cancer Registry Annual Report are varied in each year, e.g., in our study, the number of county-level registries increased from 20 to 49 between 2006 and 2009 and from 106 to 276 between 2010 and 2014. Initially, from 2006 to 2014, there were 309 county-level registries in total covered by the Chinese Cancer Registry Annual Report. The 295 county-level registries were selected, primarily because we could collect the comprehensive socioeconomic data. These cancer registries are located in 31 of 34 Chinese provinces, autonomous regions and municipalities provinces, covering a population of 190.21 million in 2014 (Figure 1).

### 2.2. Data Collection

#### 2.2.1. Health Outcome

The health outcome variable is the annual age-adjusted incidence rate of trachea, bronchus and lung cancers (i.e., the incidence rate of lung cancer in the following parts). This variable is defined as the number of incidence cases caused of trachea, bronchus and lung cancer per 100,000 people per year in a given county (or county-level city or district), age-adjusted using Segi’s world population. Health outcome data were separately reported for males and females. Hence, the original health outcome data used in the present study have excluded the effects of age and sex on health outcome.

**Figure 2:**
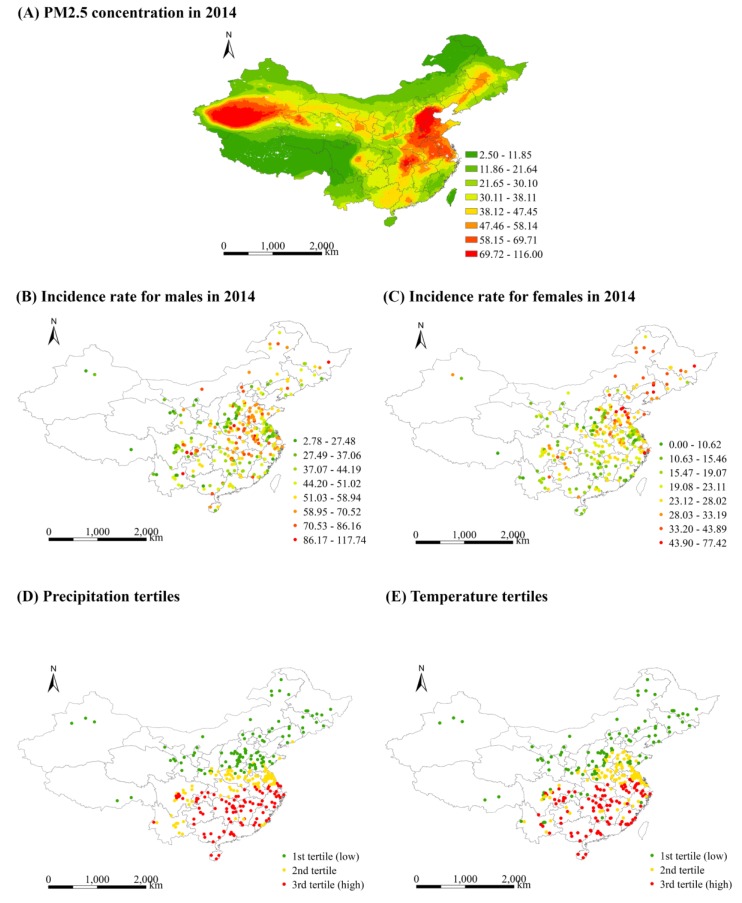
Spatial distribution of PM2.5, lung cancer outcome, and modifiers of temperature and precipitation.

Our health outcome data were collected from the Chinese Cancer Registry of the National Cancer Centre, led by the Disease Prevention and Control Bureau, Ministry of Health, China. The National Cancer Centre in China releases the Chinese Cancer Registry annual reports every year, which are considerably comprehensive and representative at the national scale. For example, the 2017 Chinese Cancer Registry annual report released the data of specific cancer incidence and mortality for 339 cancer registries in 2014, which covered 31 of 34 Chinese provinces, autonomous regions and municipalities, and a population of more than 288 million in China [4]. In terms of the International Classification of Diseases (ICD) version 10 (ICD-10), data on the annual age-adjusted incidence rate of trachea, bronchus and lung cancers (C33–C34) from 2006 to 2014, were extracted from the Chinese Cancer Registry annual reports in 2009–2017. Figure 2B,C show the spatial distribution of the incidence rate of lung cancer for males and females in 2014, respectively.

#### 2.2.2. Air Pollution

The variable of air pollution is the annual mean PM2.5 concentration in each county (or district). PM2.5 data from 2006 to 2014 (to examine concurrent effect of PM2.5) were collected from the dataset of Global Annual PM2.5 Grids from MODIS, MISR and SeaWiFS Aerosol Optical Depth (AOD) with GWR, v1 (1998–2016), released by the Socioeconomic Data and Applications Center, NASA (http://beta.sedac.ciesin.columbia.edu/data/set/sdei-global-annual-gwr-pm2-5-modis-misr-seawifs-aod). In this dataset, multiple satellite instruments of the NASA Moderate Resolution Imaging Spectroradiometer (MODIS), the Multi-Angle Imaging Spectroradiometer (MISR), and the Sea-Viewing Wide Field-of-View Sensor (SeaWiFS) were combined to retrieve aerosol optical depth (AOD). Then, a GEOS-Chem chemical transport model was employed to relate the retrieved AOD to near-surface PM2.5 concentrations to produce the annual global time series of PM2.5 concentration with nearly 1 km × 1 km resolution from 1998 to 2016 [20]. Considering the residual PM2.5 bias in the initial satellite-derived values, together with the global ground-based measurements, a geographically weighted regression model was developed to adjust for such bias. It has been shown that there was high consistency between the satellite-derived ground-level PM2.5 data and monitored measurements with R^2^ = 0.81 [21]. To date, this dataset has been widely employed to assess population exposure to PM2.5 [22,23,24]. Figure 2A presents the spatial distribution of PM2.5 concentration in 2014.

#### 2.2.3. Weather Condition

The variables of weather condition are the mean temperature and precipitation in each county. Due to the potential modification effects indicated in some studies [25,26], the mean temperature and precipitation were selected to examine whether weather factors modify the association between air pollution and the incidence rate of lung cancer in the present study. The temperature and precipitation data were collected from the UDel_AirT_Precip dataset, published by the Earth System Research Laboratory at National Oceanic and Atmospheric Administration (NOAA), USA (https://www.esrl.noaa.gov/psd/data/gridded/data.UDel_AirT_Precip.html). This dataset was mainly drawn from the Global Historical Climatology Network (GHCN2) and Legates and Willmott’s station records of monthly and annual mean air temperature and total precipitation; it provided the data of monthly time series of surface air temperature and precipitation with approximately 50 km × 50 km resolution from 1990 to 2014 [27]. To date, the UDel_AirT_Precip dataset has been widely used to characterise climate patterns and estimate climate effects [28,29].

#### 2.2.4. Socioeconomic Characteristics, Location and Time Covariates

Socioeconomic data for each county (or district), from 2006 to 2014, were collected from multiple sources: (1) China County (City) Economic Statistical Yearbook; (2) tabulation of the 2010 population census of the People’s Republic of China; (3) Report on the Work of the Government and (4) Statistical Communique on National Economic and Social Development. As in many studies adjusting for the socioeconomic factors that are related to lung cancer [14,30,31,32,33], we included the socioeconomic indicators of finance per capita, education level (i.e., average education years), percentage of construction workers, population size and urban-rural dummy. Finance per capita and education level represent the economic and education condition-induced differences, respectively, in lung cancer incidence across counties (or districts). The percentage of construction workers represents the occupation-induced differences. Population size and urban-rural dummy serve as confounders that represent the comprehensive measures of socioeconomic differences in health outcome.

To control for the effects of time and location, as in many studies [34,35], we added the degrees of longitude and latitude and a dummy variable for year into our regression models.

#### 2.2.5. Health and Behaviour Covariates

We extracted the health and behaviour data from the 2015 China Health and Retirement Longitudinal Study (CHARLS) wave4, publicly released by the National School of Development of Peking University (http://charls.pku.edu.cn/en/page/data/2015-charls-wave4). Briefly, as a high quality nationally representative survey of Chinese residents with ages 45 or older, CHARLS aims to evaluate the socioeconomic and health situations of Chinese residents. The CHARLS wave4 included about 12,400 households and 23,000 individuals, which are located in 28 of 34 Chinese provinces, autonomous regions and municipalities. Based on the module of health status and functioning within the CHARLS survey, we extracted and calculated the health-related covariates of smoking, number of cigarettes smoked per day, alcohol consumption, hypertension and diabetes.

### 2.3. Statistical Analysis

Two multivariable linear regression models were employed to estimate the effects of concurrent PM2.5 exposure on the annual incidence rates of lung cancer. Model 1 only included the annual mean concurrent PM2.5 concentration, time and location factors. Model 2 adjusted for the socioeconomic characteristics including finance per capita, education level (i.e., average education years), the percentage of construction workers, population size and urban-rural dummy variable. These model specifications are to not only mitigate the effects of unmeasured or unobserved county-specific covariates (e.g., socioeconomic characteristics), but also address the impacts of time and location. Notably, with respect to the estimate of PM2.5 effect in the two models, we treated the mean incidence rate of counties (districts) with the lowest PM concentrations as the baseline for comparison. To examine the lagged effects of PM2.5, we used not only single-year lags (same year (lag0), 1 year prior (lag1)…8 year prior (lag8)) but also moving average-year lags (lag0–1, lag0–2…lag0–8) in terms of the two multivariable linear regression models that were employed in previous estimations.

Furthermore, two sensitivity analyses were conducted to examine the robustness of concurrent PM2.5 effects. First, we examined whether our estimates were sensitive to the control of health and behaviour factors. These covariates included smoking, number of cigarettes smoked per day, alcohol consumption, hypertension and diabetes. Notably, since active smoking contributed the most to lung cancer burden in Chinese males [36], we incorporated the male smoking prevalence into the analysis for the male group. As for females, since second-hand smoking is mainly responsible for the effects of smoking on female lung cancer [36], we incorporated the total smoking prevalence into the analysis for the female group. We restricted our samples to those that are located in the targeted cities of the CHARLS survey. Hence, there were about half of the original samples left in our robust analysis. Since the information of individual location is available at the prefecture-level city, counties (or districts) that are located in the same prefecture-level city were attributed with same health and behaviour information. Second, because of the issue of changing cancer registries in each year, we estimated PM2.5 effects using data from 91 registries with consecutive 5 year records.

Finally, we investigated the potential modification effects of region, temperature and precipitation on the association between concurrent exposure to PM2.5 and health outcome. The Qin Mountains-Huai River line (Figure 1) was selected to divide counties into northern and southern counties. There are great differences in climate and human lifestyle across the Qin Mountains-Huai River boundary [37], which are key determinants of human health. In addition, it has been suggested that China’s Huai River Policy has led to differential air pollution levels and health effects between northern and southern cities [35,38]. We also investigated the potential modification effects of weather factors (i.e., temperature and precipitation), which has been indicated in some recent studies [25,26]. First, data were stratified according to the tertile divisions of weather modifiers. Then, we combined the stratified data and added the interactions between concurrent PM2.5 and modifier dummies to Model 2. Due to the modifier dummy variable’s high correlation with not only PM2.5 but also its interaction term, we did not include the modifier dummy variable in Model 2.

## 3. Results

### 3.1. Descriptive Statistics

Table 1 provides the summary statistics for data on the annual age-adjusted incidence rates of lung cancers for males and females, air pollution concentrations, weather conditions and socioeconomic characteristics. The mean incidence rates of lung cancer for males and females were 50.38 and 22.16 per 100,000 people, respectively. In parallel, the incidence rates of lung cancer for both males and females dramatically differed across counties/districts with the standard deviations of 17.15 and 8.89 for males and females, respectively. For air pollution concentration, the annual mean PM2.5 also greatly varied among counties with values of 43.02 μg/m^3^. The annual average temperature and precipitation were 14.12 °C and 8.11 mm and also considerably varied among counties (districts). Similarly, we also observed differences in socioeconomic characteristics.

### 3.2. Association between PM2.5 and the Incidence Rates of Lung Cancer for Males and Females

Table 2 and Table 3 present the estimates of concurrent PM2.5 effects on the incidence rates of lung cancer for males and females in the same year. In Model 1, if PM2.5 changes by 10 μg/m^3^, then the incidence rate relative to its baseline significantly changes by 4.83% (95% CI: 3.36%, 6.09%) and 2.07% (95% CI: 0.83%, 3.81%) for males and females, respectively. When further controlling for socioeconomic characteristics in Model 2, a 10 μg/m^3^ change in PM2.5 was still significantly and positively associated with a 4.20% (95% CI: 2.73%, 5.88%) change in the incidence rate relative to its baseline for males (Table 2). Similarly, the female incidence rate relative to its baseline also significantly changes by 2.48% (95% CI: 1.24%, 4.14%) if there was a 10 μg/m^3^ change in PM2.5 (Table 3).

**Figure 3:**
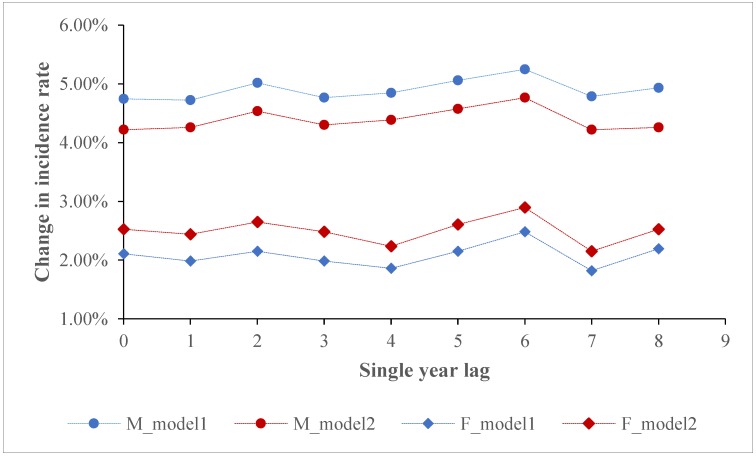
Association between PM2.5 and the incidence rates of lung cancer using different single year lags.

**Figure 4:**
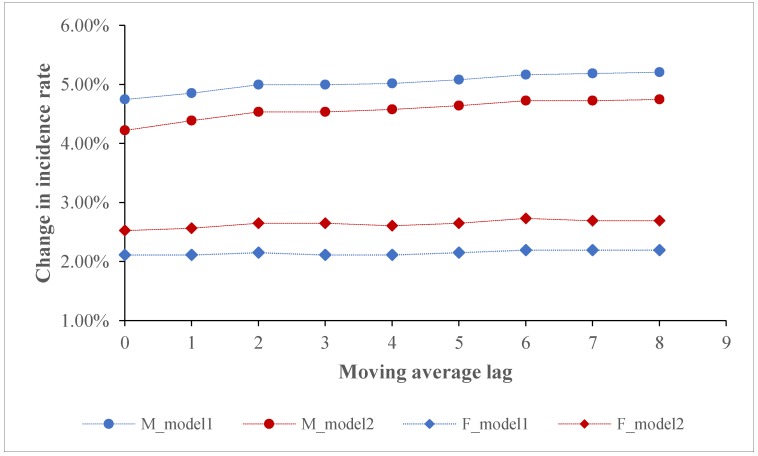
Association between PM2.5 and the incidence rates of lung cancer using different moving average year lags.

Figure 3 and Figure 4 show the long-term lag effects of PM2.5 on the incidence rates of lung cancer for males and females in the two models. With respect to the single year lag, the effects of PM2.5 on the incidence rates of lung cancer were all significant. Meanwhile, there was a slight fluctuation in the size of PM2.5 effect with the largest effect size occurring on lag6 for males and females. Regarding the moving average year lag, there were significant effects of PM2.5 on incidence rates of lung cancer at all moving average year lags. We found that among the moving average year lags, the exposure to concurrent PM2.5 had the smallest effects on incidence rates of lung cancer for males. With an elevation of moving average year lags, PM2.5 effects increased but then levelled off for males, while the effects on incidence rate of lung cancer for females almost remained unchanged.

### 3.3. Sensitivity Analysis

#### 3.3.1. Adjustment of Health and Behaviour Covariates

Sensitivity analysis of PM2.5 effects adjusted by health and behaviour covariates is shown in Table 4 and Table 5. In general, the estimates of PM2.5 effects on the incidence rates of lung cancer for both males and females were robust to the further adjustment of health and behaviour covariates. As for males, if PM2.5 changes by 10 μg/m^3^, then the incidence rate relative to its baseline significantly changes by 4.54% (95% CI: 2.80%, 6.29%) and 6.29% (95% CI: 4.54%, 8.04%) without and with adjustments (Table 4), respectively. It can also be observed that smoking status, cigarettes per day (i.e., smoking intensity) and alcohol consumption had significant and positive effects on the incidence rate of lung cancer for males (Table 4). A similar pattern of results was observed for females (Table 5).

#### 3.3.2. Issue of the Changing Cancer Registries in Each Year

Table 6 presents the sensitivity analysis of PM2.5 effects for the issue of the changing cancer registries in each year. Overall, the estimates of PM2.5 effects were not sensitive to the changing cancer registries in each year. More specifically, when using 5 year consecutive data from 91 counties, despite the slight variation in PM2.5 effect size, there still exists a significant association between concurrent PM2.5 concentration and the incidence rate of lung cancer for both males and females in the same year (Table 6). That is, a 10 μg/m^3^ change in the concurrent PM2.5 was still positively associated with a 5.67% (95% CI: 3.05%, 8.29%) and 1.72% (95% CI: −0.86%, 4.29%) change in the incidence rate relative to its baseline for males and females, respectively.

### 3.4. Modification Effects of Region, Temperature and Precipitation

The modification effect of region on the association between concurrent PM2.5 and the incidence rates of lung cancer was shown in Table 7. In general, we did not find a significant modification effect with respect to region. More specifically, we found that the interaction between PM2.5 and the region dummy variable was not significantly associated with the incidence rate of lung cancer for males (Table 7). A similar pattern of results was found for females. That is, there was no significant effect of the interaction between PM2.5 and the region dummy variable on female incidence rate (Table 7).

As shown in Table 8, temperature appeared to positively modify the effect of PM2.5 on the incidence rate of lung cancer for males. More specifically, if there was a 10 μg/m^3^ change in PM2.5, then the change in male incidence rate relative to its baseline was significantly higher by 1.47% (95% CI: 0.01%, 2.94%) and 3.99% (95% CI: 2.10%, 5.67%) in the middle and high temperature groups than in the low temperature group, respectively (Table 8). By contrast, with respect to females, we did not find a significant modifying role of temperature. That is, the interaction between PM2.5 and the temperature dummy variable was not significantly correlated with the female incidence rate (Table 8). Table 9 presents the effect modification by precipitation. In general, there was no significant modification effect of precipitation. We found that the interactions between PM2.5 and the precipitation dummy variable were not all significantly associated with the incidence rate of lung cancer for males (Table 9). A similar pattern of results was observed for females (Table 9).

## 4. Discussion

It is essential to systematically examine air pollution-associated health effects in developing countries where the air pollution level is considerably higher than that in developed countries. However, studies that estimate the effect of PM2.5 on lung cancer incidence in developing countries are still quite limited. In addition, cross-sectional studies that show PM2.5 effects which might not be visible in the individual or cohort studies are also quite limited. Cross-sectional studies, in combination with cohort studies, would contribute to an in-depth understanding of PM2.5 effects on lung cancer incidences.

To our knowledge, this is one of the few nationwide studies that showed positive associations between long-term exposure to PM2.5 and annual incidence rates of lung cancer for both males and females in China. We determined the long-term lag effects of PM2.5 on the incidence rates for both Chinese males and females. Our estimate of the PM2.5 effect was robust to not only the control socioeconomic characteristics, but also the further adjustments of health and behaviour covariates. More fundamentally, we found some evidence of effect modification by temperature.

This study estimated that if PM2.5 changes by 10 μg/m^3^, then the incidence rates of lung cancer relative to its mean (for comparison, the mean value was used as baseline) changes by 3.57% and 2.71% for males and females, respectively, which might be considerably lower than those reported in developed countries. In particular, a multisite study of 100 counties in North Carolina indicated that a 10 μg/m^3^ change in PM2.5 was positively associated with a 17.28% change in the incidence rate of lung cancer relative to its mean [39], which is considerably higher than our estimates. The lower effect size of PM2.5 found in our long-term study was also found in the short-term studies in China. As suggested in a time-series study conducted in 272 representative Chinese cities from 2013 to 2015, the effect of short-term exposure to PM2.5 on cardiopulmonary-caused mortality in China was lower than those reported in developed counties [19]. However, since cross-sectional studies using the incidence rate of lung cancer as the health outcome are still quite limited and it is also hard to compare our estimates with those from cohort studies because of the difference in study design and estimation method [9,30], more studies are required to confirm the lower PM2.5 effect found in our study. One possible explanation for a lower PM2.5 effect in China might come from the difference in PM2.5 composition. PM2.5 composition in China has a high proportion of crustal materials and dust [40], which are less toxic than the fossil-combustion-associated PM2.5 component [41,42]. The second explanation might be the tremendous difference in aging situation between China and Western Countries. Despite a rapid aging process in China at the present, the proportion of ageing people (people aged 65 and over) in China was 8.2% in 2010, which is still significantly lower than 13.6% and 20.60% in the US and Germany [43], making Chinese people less sensitive to exposure to PM2.5.

We found the long-term lag effects of PM2.5 on lung cancer incidences, which was seldom reported in previous studies. In the present study, PM2.5 with different single year lags (lag0 to lag8) or moving average year lags ((lag0–1) to (lag0–8)) were all significantly associated with the incidence rate of lung cancer for both males and females. This finding was consistent with that from similar studies. For example, a multisite study of 72 Chinese cities indicated that both the concurrent and previous 8 year annual PM2.5 concentrations were correlated with lung cancer incidence for males [15]. A nationwide population-based multigenerational panel study from 1990 to 2014 in the US suggested that prenatal and early postnatal exposure to PM2.5 was positively correlated with Childhood Asthma [44]. Findings from other similar studies further enhanced the evidence of long-term lag effects of air pollution in China [45,46] as well as western countries [30].

We found that with an elevation of moving average-year lags, PM2.5 effects on the incidence rate of lung cancer for males increased but finally levelled off. The stronger effects that occurred before the level-off point might stem from the fact that with long-term exposure to PM2.5, people tend to intake a low dose of PM2.5; the low intake then causes a slow and subtle change in human lung function, which increasingly leads to a stronger impact on lung cancer incidence. Empirically, the increasingly stronger lag effects could be also found in both the short-term and long-term studies. In particular, a recent time-series study in Shanghai indicated that with an elevation of PM2.5 moving average-day lags (lag01–lag05), the size of PM2.5 effect on daily outpatient visits increased [47]. Similarly, a time-series study performed in Saudi Arabia also found that a larger PM2.5 moving average-day lag was associated with a greater cardiovascular visits risk for males, females and the combined groups [48]. Another short-term study that examined the effect of PM2.5 with different lags on respiratory hospital admissions further enhanced the evidence of PM2.5 lag effects [49]. In addition, a long-term study suggested that compared with the concurrent PM exposure, the second and third-year exposures to PM after birth (i.e., PM1, PM2.5 and PM10) exerted stronger effects on autism spectrum disorder for children [50]. The findings in the present study highlight that lung cancer incidence is correlated with not only current PM2.5 exposure, but also the previous exposure to PM2.5. Moreover, the estimates by exclusively relying on concurrent PM2.5 exposure might underestimate the actual PM2.5-associated health effects.

We found a positive effect modification by temperature. Despite a recent increasing interest in examining the modification effect of temperature, the findings are mixed. While some short-term studies suggested that the higher temperature was correlated with a strong relationship between air pollution and health outcomes [51,52], others found a negative effect modification by temperature [53,54]. Our findings support the former argument. The mechanisms of how temperature modifies the association between PM2.5 and health outcomes remain unclear. One possible explanation might be the changed exposure to PM2.5. Temperature can exert effects on the surface air quality by way of affecting the ventilation and dilution of air pollution and the response in atmospheric chemistry [55]. A cold climate may result in restricted outdoor activities and less efficient penetration of PM2.5 from outdoors to indoors, thus leading to larger measurement errors in colder regions than in warmer regions when using the outdoor fixed-site monitoring data.

Several limitations and future work should be discussed in our study. First, with respect to the sensitivity analysis of PM2.5 effects adjusted by health and behaviour covariates, we attributed the same health-related covariates to counties (districts) that are located in the same prefecture-level city, which might ignore the behaviour variation between these counties. Second, as in many studies that examined the concurrent, 2, 3 or 8 year lag effects of PM2.5 on lung cancer [14,15,56], the 8 year lag (both single and moving average) employed in the present study might be still too short to account for the potential long-latency of lung cancer. We hope to address this limitation in the future if PM2.5 data prior to 1998 become available. Third, as in most previous ecological studies, there are inevitable errors in exposure measurement in our study (i.e., the mean PM2.5 concentration in a county to proxy exposure), because both air pollution variation and the pattern of daily human activity could affect actual human exposure [57,58]. Fourth, cohort studies which can relate individual health outcome to PM2.5 exposure are highly required in the future to test our findings because of the ecological nature in the present study.

## 5. Conclusions

Long-term exposure to PM2.5 was positively associated with the incidence rate of lung cancer for both males and females in China. The estimated effect sizes might be lower than those reported in developed countries. There were long-term lag effects of PM2.5 on the incidence rates of lung cancer for both males and females in China.

## Figures and Tables

**Table 1:** Descriptive statistics of health outcomes, air pollution, weather and socioeconomic covariates.

Variables	Mean	SD	Min	First Quartile	Median	Third Quartile	Max
Health outcomes (per 100,000 people)							
Incidence rate of lung cancer (males)	50.38	17.15	0.00	38.63	49.35	60.39	125.51
Incidence rate of lung cancer (females)	22.16	8.89	0.00	16.02	20.79	26.92	77.42
Air pollution							
PM2.5 (μg/m^3^)	43.02	16.50	2.56	30.66	44.70	55.83	87.42
Weather condition							
Precipitation (mm)	8.11	3.80	0.40	5.25	7.37	10.41	20.45
Temperature (°C)	14.12	4.60	−4.95	12.50	15.35	16.71	23.52
Socioeconomic characteristics							
Finance per capita	31.08	38.51	0.76	11.12	24.24	42.44	937.88
Average education years_male (years)	9.35	1.02	7.28	8.74	9.12	9.56	13.39
Average education years_female(years)	8.45	1.26	5.16	7.66	8.17	8.81	12.64
Percentage of construction workers	0.33%	0.24%	0.04%	0.19%	0.28%	0.43%	3.14%
Population size	68.85	33.88	4.00	39.63	64.00	92.83	188.09

**Table 2:** Association between concurrent PM2.5 and the incidence rate of lung cancer for males.

Male Baseline Rate = 47.63				
	Model 1		Model 2	
Variables	β	95% CI	β	95% CI
PM2.5	4.83% ***	(3.36%, 6.09%)	4.20% ***	(2.73%, 5.88%)
Log	0.22 ***	(0.05, 0.38)	0.30 ***	(0.14, 0.47)
Lat	−0.06	(−0.25, 0.14)	−0.06	(−0.26, 0.14)
Year 2007	4.96	(−4.95, 14.87)	4.69	(−5.03, 14.41)
Year 2008	6.17	(−3.64, 15.99)	5.78	(−3.85, 15.41)
Year 2009	6.86	(−1.74, 15.47)	6.15	(−2.30, 14.60)
Year 2010	9.58 **	(1.67, 17.49)	8.22 **	(0.42, 16.01)
Year 2011	11.80 ***	(3.99, 19.61)	10.50 ***	(2.76, 18.24)
Year 2012	14.54 ***	(6.77, 22.3)	13.53 ***	(5.77, 21.28)
Year 2013	12.87 ***	(5.25, 20.48)	12.09 ***	(4.57, 19.61)
Year 2014	13.53 ***	(5.99, 21.06)	12.30 ***	(4.85, 19.76)
Finance			−0.08	(−0.37, 0.20)
Education			−0.37	(−1.86, 1.11)
Construction			−0.40	(−0.92, 0.12)
Population			0.03 **	(0.00, 0.07)
Urban-rural			8.49 ***	(5.15, 11.83)

** for *p* < 0.05, and *** for *p* < 0.01. With a 10 μg/m^3^ change in PM2.5, the change in the incidence rate relative to its baseline = (10 × coefficient for PM2.5)/baseline incidence rate (i.e., 47.63 per 100,000 people).

**Table 3:** Association between concurrent PM2.5 and the incidence rate of lung cancer for females.

Female Baseline Rate = 24.17				
	Model 1		Model 2	
Variables	β	95% CI	β	95% CI
PM2.5	2.07% ***	(0.83%, 3.31%)	2.48% ***	(1.24%, 4.14%)
Log	0.19 ***	(0.11, 0.26)	0.23 ***	(0.15, 0.31)
Lat	0.54 ***	(0.45, 0.63)	0.52 ***	(0.42, 0.61)
Year 2007	0.93	(−3.84, 5.69)	0.82	(−3.83, 5.48)
Year 2008	0.66	(−4.06, 5.37)	0.39	(−4.22, 5.01)
Year 2009	2.86	(−1.28, 6.99)	2.58	(−1.47, 6.63)
Year 2010	3.85 **	(0.04, 7.65)	3.15 *	(−0.59, 6.88)
Year 2011	4.78 **	(1.03, 8.53)	4.05 **	(0.34, 7.76)
Year 2012	6.5 ***	(2.77, 10.23)	5.8 ***	(2.08, 9.51)
Year 2013	6.18 ***	(2.52, 9.84)	5.47 ***	(1.87, 9.08)
Year 2014	7.04 ***	(3.42, 10.66)	6.18 ***	(2.61, 9.75)
Finance			0.02	(−0.11, 0.16)
Education			−0.44	(−1.02, 0.14)
Construction			−0.76 ***	(−1.01, −0.52)
Population			0.01	(−0.01, 0.02)
Urban-rural			3.26 ***	(1.68, 4.85)

* for *p* < 0.1, ** for *p* < 0.05, and *** for *p* < 0.01. With a 10 μg/m^3^ change in PM2.5, the change in the incidence rate relative to its baseline = (10 × coefficient for PM2.5)/baseline incidence rate (i.e., 24.17 per 100,000 people).

**Table 4:** Sensitivity analysis of PM2.5 effect adjusted by health and behaviour covariates for males.

Male Baseline Rate = 57.24				
	Without Control		Control	
Variables	β	95% CI	β	95% CI
PM2.5	4.54% ***	(2.80%, 6.29%)	6.29% ***	(4.54%, 8.04%)
Log	0.29 **	(0.02, 0.56)	0.65 ***	(0.34, 0.96)
Lat	−0.06	(−0.36, 0.24)	0.09	(−0.24, 0.42)
Year 2007	6.9	(−5.54, 19.33)	7.14	(−4.8, 19.07)
Year 2008	5.31	(−7.12, 17.74)	6.74	(−5.2, 18.68)
Year 2009	6.76	(−4.45, 17.97)	7.34	(−3.43, 18.11)
Year 2010	13.65 ***	(3.35, 23.96)	12.79 **	(2.87, 22.71)
Year 2011	13.66 ***	(3.44, 23.88)	13.05 ***	(3.22, 22.88)
Year 2012	15.56 ***	(5.34, 25.78)	15.7 ***	(5.86, 25.53)
Year 2013	15.26 ***	(5.37, 25.15)	14.32 ***	(4.8, 23.83)
Year 2014	14.37 ***	(4.58, 24.15)	13.74 ***	(4.33, 23.15)
Finance	0.16	(−0.13, 0.46)	0.07	(−0.22, 0.35)
Education	3.05 ***	(1.02, 5.09)	2.26 **	(0.25, 4.27)
Construction	−0.12	(−0.84, 0.6)	−0.41	(−1.12, 0.3)
Population	−0.01	(−0.06, 0.03)	−0.02	(−0.07, 0.02)
Urban-rural	3.74	(−1.09, 8.56)	4.65 **	(−0.23, 9.53)
Smoking status			29.2 ***	(16.72, 41.68)
Cigarettes per day			0.39 ***	(0.12, 0.67)
Hypertension			−12.9	(−36.8, 11)
Diabetes			−11.53	(−57.12, 34.07)
Alcohol			55.66 ***	(31.1, 80.23)

** for *p* < 0.05, and *** for *p* < 0.01. With a 10 μg/m^3^ change in PM2.5, the change in incidence rate relative to its baseline = (10 × coefficient for PM2.5)/baseline incidence rate (i.e., 57.24 per 100,000 people).

**Table 5:** Sensitivity analysis of PM2.5 effect adjusted by health and behaviour covariates for females.

Female Baseline Rate = 31.83				
	Without Control		Control	
Variables	β	95% CI	β	95% CI
PM2.5	3.14% ***	(1.57%, 4.71%)	3.77% ***	(2.20%, 5.66%)
Log	0.24 ***	(0.11, 0.38)	0.28 ***	(0.14, 0.41)
Lat	0.44 ***	(0.29, 0.59)	0.52 ***	(0.35, 0.69)
Year 2007	−1.25	(−7.43, 4.94)	−1.05	(−7.18, 5.08)
Year 2008	−1.93	(−8.11, 4.26)	−1.61	(−7.74, 4.52)
Year 2009	0.79	(−4.79, 6.36)	1.06	(−4.47, 6.6)
Year 2010	3.19	(−1.93, 8.31)	3.33	(−1.78, 8.44)
Year 2011	3.58	(−1.5, 8.66)	3.92	(−1.15, 8.99)
Year 2012	4.88 **	(−0.21, 9.96)	5.38 **	(0.3, 10.46)
Year 2013	4.15 *	(−0.77, 9.07)	4.34 *	(−0.58, 9.25)
Year 2014	4.85 **	(−0.01, 9.72)	5.14 **	(0.26, 10.02)
Finance	0.06	(−0.08, 0.21)	0.05	(−0.1, 0.19)
Education	0.64	(−0.17, 1.45)	0.31	(−0.56, 1.17)
Construction	−0.48 ***	(−0.83, −0.12)	−0.48 ***	(−0.84, −0.12)
Population	−0.01	(−0.03, 0.01)	−0.01	(−0.03, 0.01)
Urban-rural	1.59	(−0.75, 3.93)	1.87	(−0.5, 4.24)
Smoking status			19.21 **	(2.55, 35.87)
Cigarettes per day			0.15 **	(0.01, 0.29)
Hypertension			−7.52	(−21.72, 6.67)
Diabetes			5.86	(−23.73, 35.45)
Alcohol			19.1 **	(−1.34, 39.53)

* for *p* < 0.1, ** for *p* < 0.05, and *** for *p* < 0.01. With a 10 μg/m^3^ change in PM2.5, the change in incidence rate relative to its baseline = (10 × coefficient for PM2.5)/baseline incidence rate (i.e., 31.83 per 100,000 people).

**Table 6:** Sensitivity analysis using 5 year consecutive data from 91 counties for males and females.

	Male		Female	
	Male Baseline Rate = 45.85		Female Baseline Rate = 23.32	
Variables	β	95% CI	β	95% CI
PM2.5	5.67% ***	(3.05%, 8.29%)	1.72% *	(−0.86%, 4.29%)
Log	−0.02	(−0.3, 0.27)	0.1	(−0.03, 0.24)
Lat	−0.45 ***	(−0.76, −0.14)	0.36 ***	(0.21, 0.51)
Year 2011	1.26	(−3.21, 5.74)	0.68	(−1.48, 2.84)
Year 2012	3.97 *	(−0.64, 8.58)	2.01 *	(−0.22, 4.23)
Year 2013	3.21	(−1.23, 7.65)	2.33 **	(0.19, 4.47)
Year 2014	3.5	(−0.96, 7.96)	2.02 **	(−0.13, 4.17)
Finance	−0.15	(−0.46, 0.16)	−0.07	(−0.22, 0.08)
Education	−1.62	(−3.71, 0.46)	−0.51	(−1.33, 0.3)
Construction	−1.02 **	(−1.84, −0.2)	−0.6 ***	(−1, −0.2)
Population	0.08 ***	(0.02, 0.13)	0.03 **	(0.01, 0.06)
Urban-rural	9.98 ***	(5.38, 14.57)	2.51	(0.35, 4.67)

* for *p* < 0.1, ** for *p* < 0.05, and *** for *p* < 0.01. With a 10 μg/m^3^ change in PM2.5, the change in incidence rate relative to its baseline = (10 × coefficient for PM2.5)/baseline incidence rate (i.e., 45.85 and 23.32 per 100,000 people for males and females, respectively).

**Table 7:** Modification effects of region (i.e., North vs. south).

	Male		Female	
	Male Baseline Rate = 47.63		Female Baseline Rate = 24.17	
Variables	β	95% CI	β	95% CI
PM2.5	4.20% ***	(2.31%, 6.09%)	1.65% **	(0.00%, 3.31%)
Log	0.30 ***	(0.14, 0.47)	0.25 ***	(0.16, 0.33)
Lat	−0.06	(−0.3, 0.18)	0.44 ***	(0.33, 0.56)
Year 2007	4.69	(−5.03, 14.42)	0.91	(−3.73, 5.56)
Year 2008	5.79	(−3.85, 15.42)	0.45	(−4.16, 5.05)
Year 2009	6.15	(−2.3, 14.6)	2.61	(−1.43, 6.65)
Year 2010	8.21 **	(0.41, 16.01)	3.07	(−0.65, 6.8)
Year 2011	10.49 ***	(2.75, 18.24)	3.93 **	(0.22, 7.63)
Year 2012	13.52 ***	(5.75, 21.28)	5.63 ***	(1.92, 9.34)
Year 2013	12.09 ***	(4.56, 19.61)	5.35 ***	(1.75, 8.94)
Year 2014	12.3 ***	(4.84, 19.76)	6.06 ***	(2.49, 9.62)
Finance	−0.08	(−0.37, 0.2)	0.04	(−0.1, 0.18)
Education	−0.37	(−1.87, 1.13)	−0.39	(−0.97, 0.19)
Construction	−0.4	(−0.92, 0.13)	−0.72 ***	(−0.97, −0.47)
Population	0.03 **	(0, 0.07)	0.01	(−0.01, 0.02)
Urban-rural	8.48 ***	(5.12, 11.84)	3.11 ***	(1.53, 4.7)
PM2.5 × North	0.04%	(−1.05%, 1.26%)	1.24%	(0.00%, 2.48%)

** for *p* < 0.05, and *** for *p* < 0.01. With a 10 μg/m^3^ change in PM2.5, the change in incidence rate relative to its baseline = (10 × coefficient for PM2.5 or its interaction terms)/baseline incidence rate (i.e., 47.63 and 24.17 per 100,000 people for males and females, respectively).

**Table 8:** Modification effects of temperature.

	Male		Female	
	Male Baseline Rate = 47.63		Female Baseline Rate = 24.17	
Variables	β	95% CI	β	95% CI
PM2.5	2.10% **	(−0.21%, 4.41%)	2.48% **	(0.41%, 4.55%)
Log	0.28 ***	(0.12, 0.45)	0.24 ***	(0.16, 0.32)
Lat	0.27 **	(0.03, 0.51)	0.48 ***	(0.37, 0.6)
Year 2007	4.56	(−5.05, 14.18)	0.84	(−3.82, 5.49)
Year 2008	5.79	(−3.74, 15.31)	0.36	(−4.26, 4.97)
Year 2009	5.79	(−2.57, 14.16)	2.56	(−1.49, 6.61)
Year 2010	8.03 **	(0.3, 15.76)	3.07	(−0.67, 6.81)
Year 2011	10.24 ***	(2.56, 17.92)	3.97 **	(0.25, 7.68)
Year 2012	13.31 ***	(5.61, 21)	5.7 ***	(1.98, 9.43)
Year 2013	12.02 ***	(4.57, 19.47)	5.39 ***	(1.78, 9)
Year 2014	12.05 ***	(4.65, 19.45)	6.1 ***	(2.52, 9.68)
Finance	−0.14	(−0.42, 0.14)	0.03	(−0.1, 0.17)
Education	−0.55	(−2.05, 0.94)	−0.37	(−0.97, 0.23)
Construction	−0.51 **	(−1.03, 0)	−0.76 ***	(−1.01, −0.51)
Population	0.04 **	(0.01, 0.07)	0.01	(−0.01, 0.02)
Urban-rural	8.47 ***	(5.16, 11.79)	3.19 ***	(1.59, 4.79)
PM2.5 × Temperature2	1.47% **	(0.01%, 2.94%)	0.41%	(−1.24%, 1.65%)
PM2.5 × Temperature3	3.99% ***	(2.10%, 5.67%)	−0.83%	(−2.48%, 0.83%)

** for *p* < 0.05, and *** for *p* < 0.01. With a 10 μg/m^3^ change in PM2.5, the change in incidence rate relative to its baseline = (10 × coefficient for PM2.5 or its interaction terms)/baseline incidence rate (i.e., 47.63 and 24.17 per 100,000 people for males and females, respectively).

**Table 9:** Modification effects of precipitation.

	Male		Female	
	Male Baseline Rate = 47.63		Female Baseline Rate = 24.17	
Variables	β	95% CI	β	95% CI
PM2.5	4.83% ***	(3.15%, 6.51%)	3.31% ***	(1.65%, 4.96%)
Log	0.24 ***	(0.07, 0.4)	0.22 ***	(0.14, 0.3)
Lat	0.16	(−0.09, 0.41)	0.52 ***	(0.4, 0.64)
Year 2007	5	(−4.6, 14.59)	0.91	(−3.73, 5.55)
Year 2008	6.62	(−2.89, 16.12)	0.57	(−4.03, 5.17)
Year 2009	7.05 *	(−1.3, 15.4)	2.82	(−1.22, 6.86)
Year 2010	9.52 **	(1.81, 17.24)	3.47 **	(−0.26, 7.2)
Year 2011	11.91 ***	(4.25, 19.57)	4.34 **	(0.63, 8.04)
Year 2012	14.87 ***	(7.2, 22.54)	6.03 ***	(2.32, 9.74)
Year 2013	12.98 ***	(5.55, 20.42)	5.6 ***	(2, 9.19)
Year 2014	13.17 ***	(5.81, 20.54)	6.35 ***	(2.78, 9.91)
Finance	−0.17	(−0.45, 0.12)	0.01	(−0.12, 0.15)
Education	−0.85	(−2.33, 0.62)	−0.59 **	(−1.18, 0.01)
Construction	−0.31	(−0.84, 0.22)	−0.7 ***	(−0.96, −0.44)
Population	0.04 **	(0.01, 0.08)	0.01	(0, 0.03)
Urban-rural	8.88 ***	(5.58, 12.18)	3.49 ***	(1.91, 5.08)
PM2.5 × Precipitation2	−1.05%	(−2.31%, 0.21%)	−1.24% **	(−2.48%, 0.00%)
PM2.5 × Precipitation3	2.52% ***	(0.84%, 4.20%)	0.01%	(−1.65%, 1.65%)

* for *p* < 0.1, ** for *p* < 0.05, and *** for *p* < 0.01. With a 10 μg/m^3^ change in PM2.5, the change in incidence rate relative to its baseline = (10 × coefficient for PM2.5 or its interaction terms)/baseline incidence rate (i.e., 47.63 and 24.17 per 100,000 people for males and females, respectively).
